# Solid-State Synthesis Based on Natural Leucomisine

**DOI:** 10.3390/molecules31081307

**Published:** 2026-04-17

**Authors:** Sergazy Adekenov, Asel Amanzhan, Dmitriy Savchenko, Ivan Krasnyuk, Nataliya Solovieva, Ivan Krasnyuk

**Affiliations:** 1 JSC «Research and Production Center «Phytochemistry», M. Gazaliev St., Building 4, Karaganda 100009, Kazakhstan; arglabin@phyto.kz (S.A.); asel.amanzhan@bk.ru (A.A.); 2 Department of Analytical, Physical and Colloidal Chemistry, A.P. Nelyubin Institute of Pharmacy, Federal State Autonomous Educational Institution of Higher Education I.M. Sechenov First Moscow State Medical University of the Ministry of Health of the Russian Federation (Sechenov University), 8 Trubetskaya St., Building 2, 119991 Moscow, Russia; krasnyuk.79@mail.ru; 3 Department of Pharmaceutical Technology, A.P. Nelyubin Institute of Pharmacy, Federal State Autonomous Educational Institution of Higher Education I.M. Sechenov First Moscow State Medical University of the Ministry of Health of the Russian Federation (Sechenov University), 8 Trubetskaya St., Building 2, 119991 Moscow, Russia; soloveva_n_l@staff.sechenov.ru (N.S.); krasnyuki@mail.ru (I.K.S.)

**Keywords:** leucomisine, disodium glycyrrhizinate, solid dispersion, solubility

## Abstract

Leucomisine is a major component of renewable plant raw material *Artemisia leucodes* Schrenk, a sesquiterpene γ-lactone exhibiting antioxidant, hypoglycemic, antiparasitic, and hepatoprotective activities. However, the use of leucomisine in pharmaceuticals is limited by its insufficient bioavailability associated with low aqueous solubility. Therefore, the effect of solid-state synthesis based on leucomisine using the methods of “solvent removal”, “simple mixing”, and “mixture heating”, with disodium glycyrrhizinate as a carrier, on the aqueous solubility of leucomisine was investigated. It was established that the synthesized solid dispersions exhibit increased solubility (7–19-fold) and dissolution rate (36–100-fold) of leucomisine released from the carrier. The most pronounced stimulation of the dissolution process was observed for samples obtained using the “simple mixing” method. Based on physicochemical studies (visible-range spectrophotometry, microcrystalloscopy, investigation of optical properties of solutions, and X-Ray phase analysis), it was determined that the enhancement of solubility is attributed to the loss of crystalline state, micronization, and the solubilization process of leucomisine by the carrier, as well as to the formation of a colloidal solution of leucomisine stabilized by disodium glycyrrhizinate.

## 1. Introduction

The development of new pharmaceutical substances based on plant-derived compounds represents a promising strategy for expanding the range of original medicinal products. In the search for novel drug candidates, particular attention has been paid to the group of terpenoid compounds, specifically sesquiterpene γ-lactones with a guaiane carbon skeleton. Among guaianolides, molecules containing a dienone fragment are of special interest, as they are widely distributed in plant species of the Asteraceae and Apiaceae families. *Artemisia leucodes* Schrenk and *Artemisia austriaca* Jacq. contain up to 2.0% of cyclopentadienone guaianolides, including leucomisine (**1**), achillin (**2**), austricin (**3**), and grossmisine (**4**), which exhibit hypolipidemic and antioxidant activities [1]. One of the notable achievements in the field of medicinal chemistry of plant terpenoids is the development of a new drug based on leucomisine isolated from *Artemisia leucodes* Schrenk. Pharmacological studies have demonstrated that this compound possesses a combination of antiatherosclerotic and hypolipidemic effects [1,2].

*Artemisia leucodes* Schrenk is an annual or biennial endemic plant species native to Kazakhstan, Kyrgyzstan, Tajikistan, Turkmenistan, and Uzbekistan [3]. It is widely distributed in the southern and western parts of the Moyynkum Desert in the Zhambyl Region of the Republic of Kazakhstan, where the estimated available raw material reserves amount to 20.4 and 103 tons, respectively [4]. In Southern Kazakhstan, the exploitable reserve of dry aerial biomass over an area of 11 ha is 28 tons, with an annual harvesting volume of 11.6 tons.

The major constituent of *Artemisia leucodes* Schrenk is the guaiane-type sesquiterpene lactone leucomisine (**1**), the content of which in the aerial parts of the plant raw material ranges from 0.30% to 0.77% [5]. Leucomisine (C_15_H_18_O_3_) has a molar mass of 246.302 g/mol and a melting point of 196–198 °C.

Leucomisine was first isolated from *Artemisia leucodes* Schrenk by Prof. K. S. Rybal’ko in 1961 and subsequently from *Artemisia austriaca* Jacq. in 1963 [6,7]. Later, the isolation of this compound from *Artemisia leucodes* Schrenk was also investigated by Czech researchers in 1962 [8]. Leucomisine has additionally been identified in *Achillea millefolium* L. [9], *Artemisia juncea* [10], *Artemisia cana* ssp. viscidula Beetle, *Artemisia santolina* L., *Artemisia tridentata* ssp. *tridentata*, *Artemisia lanata* Willd., *Artemisia caucasica* Willd., *Artemisia frigida* Willd., and others.

With respect to stereochemistry, leucomisine (**1**) is classified as a non-linear guaianolide, as the lactone ring is located at C6 and C7. The stereochemistry of the five- and seven-membered carbocycles was established by total chemical synthesis [11]. The seven-membered carbocycle is trans-fused with the lactone ring, forming a dihedral angle of approximately 160–180°, which is supported by the proton chemical shifts at C-6 and C-7, exhibiting a vicinal coupling constant (^3^J) of about 10 Hz.

In the leucomisine (**1**) molecule, the cyclopentadienone and γ-lactone fragments serve as the primary reactive centers and key pharmacophoric moieties. The asymmetric centers in leucomisine (**1**) are located at carbon atoms C5, C6, C7, and C11. Accordingly, the optical activity experimentally determined using a Polax-2L polarimeter (Atago Co. LTD, Tokyo, Japan), ${\left[\alpha \right]}_{D}^{20}$ + 56° (c 2.86, chloroform), contributes to the pharmacological activity of this molecule.

Leucomisine (**1**) exhibits antiatherosclerotic and hypolipidemic effects, promoting reductions in cholesterol, triglycerides, and total lipid levels, and in this respect surpasses the reference drug pravastatin [12,13]. In a rat model of an atherogenic diet, the mechanisms underlying the hypolipidemic activity of leucomisine (**1**) in the liver were elucidated [14]. The compound was shown to increase the mRNA expression of the genes CPT1A, 3-hydroxy-3-methylglutaryl-CoA reductase (HMGCR), and carnitine palmitoyltransferase 2 (CPT2), while decreasing the expression of sterol O-acyltransferase 1 (SOAT1), an acyl-CoA: cholesterol acyltransferase.

According to the results of in vivo studies of the anthelmintic activity of leucomisine in dogs, capsules containing leucomisine at a dose of 100 mg demonstrated 100% efficacy against whipworms (*Trichuris vulpis*) and cyclophyllidean cestodes (*Dipylidium caninum*) [15]. It has been suggested that the principal enzymatic targets responsible for the anti-opisthorchiasis activity of sesquiterpene γ-lactones are thioredoxin–glutathione reductase and glutathione S-transferase. Interactions with these enzymes are believed to stabilize the molecules of cynaropicrin and grossheimin, respectively [16].

Currently, preparations such as “Leikoflan”, which exhibits corrective effects in disorders of the immune system and hepatic catabolic processes caused by radiation exposure, and “Aterolid”, which shows pronounced antiatherosclerotic and hypolipidemic activity, are produced from *Artemisia leucodes* Schrenk raw material [17].

**Figure 1:**
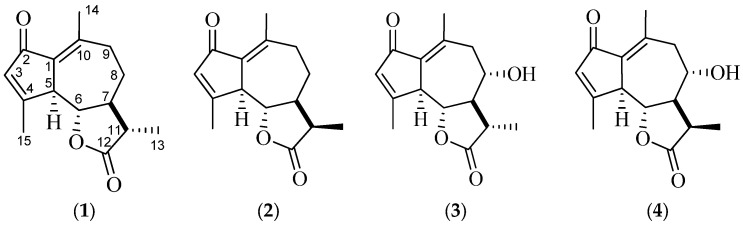
Structural formulas of compounds **1**–**4**.

The main difficulty in conducting pharmacological studies of leucomisine (Figure 1) is its practical insolubility in water and, consequently, its low bioavailability. This limitation, in some cases, restricts its application and creates technological challenges in the development of innovative medicinal products.

At present, a promising direction in pharmaceutical development is the expansion of the range of dosage forms for previously known pharmaceutically active substances [18,19]. The optimization of dissolution of active ingredients and their release from dosage forms can be achieved using the solid dispersion (SD) method [20,21]. SDs are solutions of a pharmaceutically active substance in a carrier matrix or multicomponent systems combining active agents and a carrier matrix as a solid phase dispersed in a polymer. In certain cases, the formation of various complexes between active substances and the carrier material is observed [22,23].

The concept of “solid dispersions” as a novel pharmaceutical approach was first proposed in 1961 by researchers in Japan [24]. Similar studies were subsequently conducted at the First Moscow Medical Institute (under the supervision of A. I. Tentsova) and at the Pyatigorsk Medical Institute (a branch of Volgograd State Medical University).

At present, solid dispersions (SDs) are being actively investigated at the Sechenov First Moscow State Medical University (Sechenov University), particularly at the A. P. Nelyubin Institute of Pharmacy, within the Departments of Analytical Chemistry, Physical and Colloid Chemistry, and Pharmaceutical Technology. Since 2001, extensive research carried out at Sechenov University has resulted in the detailed characterization of SDs involving more than thirty-five poorly soluble active pharmaceutical ingredients from various pharmacological groups [25].

Solid dispersions (SDs) also enhance the storage stability of pharmaceutical products by improving the resistance of the active substance to environmental factors such as moisture, light, and temperature, while enabling a uniform distribution of small amounts of the active ingredient within the solid phase [26].

Despite certain limitations, including the labor-intensive and costly preparation procedures, challenges associated with incorporation into dosage forms and control of physicochemical properties, as well as difficulties in scaling up the manufacturing process, SD technology is no longer confined to laboratory research. It is currently successfully applied on commercial platforms for the development of highly effective medicinal products [27,28].

A wide range of materials, including those of polymeric nature, are used as carrier matrices for SDs, such as hydroxypropyl methylcellulose, polyvinylpyrrolidone, silicon dioxide, and microcrystalline cellulose [29,30,31].

Thus, Chen T. et al. prepared amorphous solid dispersions of oxyberberine, a derivative of the plant alkaloid berberine, using hydroxypropyl methylcellulose acetate succinate (HPMCAS, H grade) as the carrier matrix via spray drying. The developed solid dispersions improved the oral bioavailability of the parent compound due to changes in its crystallinity. In vivo studies in mice demonstrated a significant improvement in liver function parameters and inflammatory markers compared with pure oxyberberine [32].

Researchers from the Czech Republic prepared solid dispersions based on the natural flavonoid isorhamnetin using polyvinylpyrrolidone (PVP10) and benzalkonium chloride for intravenous administration. The solubility of the parent compound in the developed solid dispersions increased by approximately 600-fold. Importantly, the pharmacological properties of isorhamnetin, including its iron-chelating and reducing activities, as well as its ability to penetrate human platelets, remained unchanged [33].

Benedikt B. et al. investigated mesoporous silicon dioxide as a carrier for poorly soluble natural compounds, including coenzyme Q10 (CoQ10) and astaxanthin (ASX), as well as poorly soluble synthetic compounds such as probucol (PB) and lumefantrine (LU). Using a biorelevant biphasic dissolution system (BiPHa+), the authors observed a pronounced increase in the equilibrium solubility of the studied substances. In particular, the natural products CoQ10 and ASX demonstrated substantial advantages when incorporated into mesoporous carrier particles, clearly outperforming currently available commercial formulations [34].

A study by Italian researchers reported a significant enhancement in the solubility and bioavailability of the synthetic nonsteroidal anti-inflammatory drug indomethacin through the preparation of microparticles containing solid dispersions with Gelucire^®^ 50/13 and 48/16 via spray congealing [35]. To further improve the solubility of indomethacin, the preparation of granulated three-component solid dispersions using urea or Kollidon^®^ VA 64 as carrier matrices and Neusilin^®^ US2 as an adsorbent has also been investigated [36].

A promising carrier for the preparation of SDs is disodium glycyrrhizinate (Na_2_GA) [37], C_42_H_60_O_16_Na_2_, a salt of glycyrrhizic acid. Glycyrrhizic acid is contained in licorice roots. In the food industry, it is used as a sweetener, and in medicine it is used as a medicinal agent (licorice preparations). In addition, it exhibits a broad spectrum of antiviral activity against viruses such as hepatitis viruses, herpes viruses, and severe acute respiratory syndrome coronavirus 2 (SARS-CoV-2), among others [38]. Its pharmacological effect is associated with the induction of interferon. Importantly, viral inhibition is observed at concentrations that are non-toxic to host cells [39]. Glycyrrhizic acid actively suppresses the replication of SARS-associated coronavirus [40,41] and inhibits the main viral protease of SARS-CoV-2 in vitro [42]. Na_2_GA is a chemically sufficiently stable substance, which is of particular importance since the data obtained in this study are intended to be used in the future for the design of rapidly dissolving dosage forms of leucomisine.

The aim of this study was to investigate the effect of solid dispersion synthesis with disodium glycyrrhizinate (Na_2_GA) on the dissolution process of leucomisine in water.

## 2. Results and Discussion

One of the objectives of this study was to identify the factors responsible for the increased solubility of leucomisine released from solid dispersions (SDs). To elucidate the mechanisms underlying the enhancement of leucomisine solubility from SDs, microcrystalloscopy, X-Ray phase analysis, and investigation of the optical properties of solutions were employed.

An analysis of the scientific literature published over the past 25 years did not reveal any reports on the use of disodium glycyrrhizinate (Na_2_GA) for the synthesis of solid dispersions aimed at improving the aqueous solubility of leucomisine. Based on experimental data and literature sources, it was determined that the optimal range of Na_2_GA: leucomisine ratios does not exceed 7:1 by mass [1,2,3,25].

The change in leucomisine solubility was calculated as the ratio of the leucomisine concentration in the saturated solution of its solid dispersion to the saturated concentration leucomisine substance solution at the end of the experiment. By the end of the experiment (60 min), the solutions of the studied samples were turbid.

**Figure 2:**
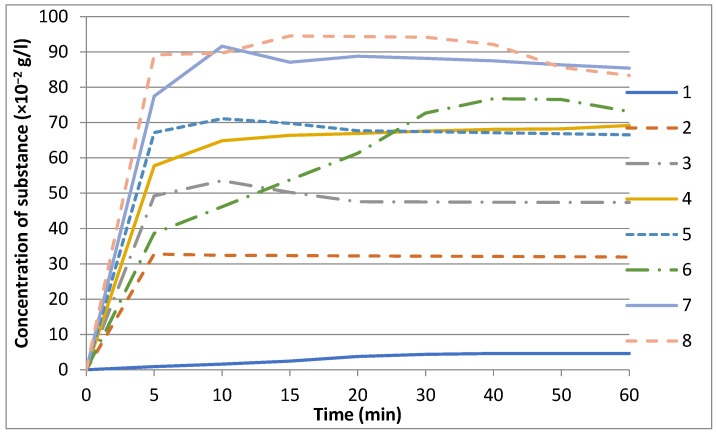
Changes in the concentration of leucomisine solutions and its solid dispersions with disodium glycyrrhizinate over time: (**1**) leucomisine (parent substance); (**2**) SD (1:2) “solvent removal”; (**3**) SD (1:3) “solvent removal”; (**4**) SD (1:5) “solvent removal”; (**5**) SD (1:7) “solvent removal”; (**6**) SD (1:5) “mixture heating”; (**7**) SD (1:5) “simple mixing”; (**8**) SD (1:7) “simple mixing”.

The initial leucomisine substance dissolves very slowly in water (Figure 2). During the experiment, the concentration of leucomisine in the solution of the parent substance changes only slightly, gradually increasing from 0.904 × 10^−2^ g/dm^3^ at 5 min to 4.602 × 10^−2^ g/dm^3^ at 60 min.

The dissolution kinetics data (Table 1 and Figure 2) demonstrate that the synthesis of solid dispersions (SDs) with Na_2_GA significantly improves both the dissolution rate of leucomisine and its aqueous solubility in all cases. It was observed that the effect of SDs on leucomisine dissolution becomes more pronounced with increasing carrier (Na_2_GA) content. Thus, with a stepwise increase in the leucomisine:Na_2_GA mass ratio in SDs prepared by the “solvent removal” method from 1:2 to 1:3 and 1:5, the solubility of leucomisine released from the SDs increases proportionally by approximately 7-, 10-, and 15-fold, respectively, reaching a level of 69.181 × 10^−2^ g/dm^3^. A further increase in the Na_2_GA content in SDs prepared by “solvent removal” to a ratio of 1:7 does not lead to a comparable enhancement of solubility; in this case, the leucomisine concentration increases by 14.5-fold to 66.559 × 10^−2^ g/dm^3^. Based on these results, a leucomisine:Na_2_GA mass ratio of 1:5 can be considered optimal for improving solubility.

A similar dependence on the Na_2_GA ratio is observed for the dissolution rate of leucomisine from SDs obtained by the “solvent removal” method. For SDs with ratios of 1:2, 1:3, 1:5, and 1:7, leucomisine dissolves during the first 5 min approximately 36-, 55-, 64-, and 74-fold faster than the parent substance, respectively, reaching a concentration of 67.175 × 10^−2^ g/dm^3^. As a result, starting from a ratio of 1:3 and higher, a supersaturation effect followed by leucomisine recrystallization is observed in the solutions of these SDs within the first 10 min of dissolution. For example, for the SD with a ratio of 1:7, beginning at 10 min (71.119 × 10^−2^ g/L) and up to 20 min (and further until the end of the experiment), the leucomisine concentration slightly decreases to an approximately constant value of ~67.000 × 10^−2^ g/dm^3^.

The method of SD preparation by “mixture heating” did not demonstrate significant advantages over the “solvent removal” or “simple mixing” methods. For the SD with a ratio of 1:5 obtained by joint heating of leucomisine and Na_2_GA, the dissolution rate and solubility of leucomisine increased by 43- and 16-fold, respectively (to 38.642 × 10^−2^ and 73.116 × 10^−2^ g/dm^3^), which does not exceed and is nearly identical to the corresponding SD of the same composition prepared by “solvent removal”. The only difference is that saturation of the solution for the SD obtained by “mixture heating” occurs more slowly, and the supersaturation phenomenon is observed much later—at approximately 40 min from the start of the experiment (maximum leucomisine concentration of 76.784 × 10^−2^ g/dm^3^).

The most pronounced effect on the dissolution process of leucomisine is exhibited by SDs prepared by the “simple mixing” method. As noted above, no significant difference is observed between the ratios of 1:5 and 1:7 in terms of their influence on the extent of leucomisine dissolution; both variants enhance it to a similar maximal degree. A comparison of SDs prepared by mixing leucomisine and Na_2_GA at ratios of 1:5 and 1:7 reveals the highest increase in solubility—18.6-fold (85.446 × 10^−2^ g/dm^3^) and 18.1-fold (83.345 × 10^−2^ g/dm^3^), respectively. These SDs enhance the dissolution rate of leucomisine in water even more markedly during the first 5–10 min—by 85.7- and 98.7-fold, respectively—allowing the highest leucomisine concentrations to be achieved over the entire experiment: 91.628 × 10^−2^ and 89.716 × 10^−2^ g/dm^3^.

**Figure A1:**
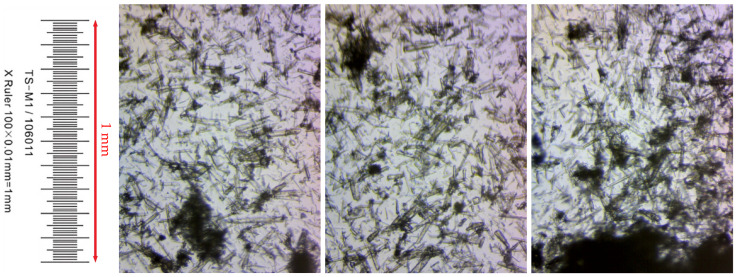
Microscopic image of the initial leucomisine (magnification ×4).

According to microscopic analysis (Appendix A), the initial leucomisine substance consists of transparent, colorless crystalline fragments of needle-like morphology. It was likely subjected to micronization at an earlier stage (Figure A1).

**Figure A2:**
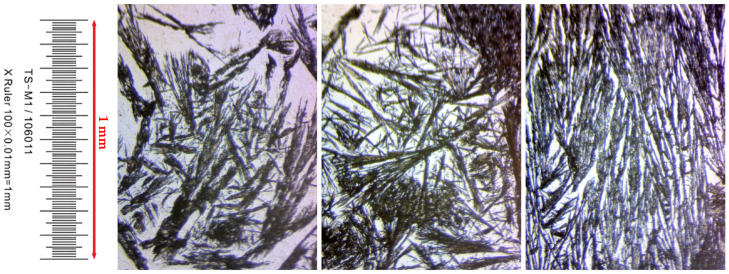
Microscopic image of recrystallized leucomisine (magnification ×4).

Leucomisine after recrystallization from an alcoholic solution (under conditions analogous to those used for SD synthesis by the “solvent removal” method) does not undergo changes in its crystalline structure; aggregates of similar transparent, needle-shaped crystals are observed (Figure A2).

**Figure A3:**
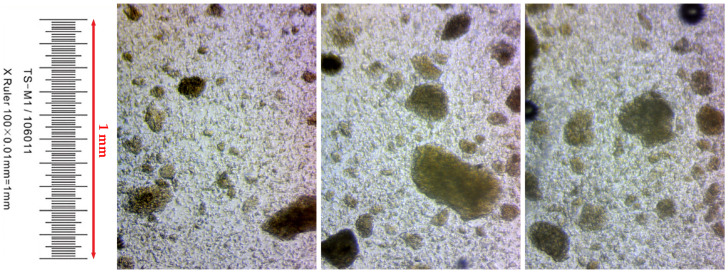
Microscopic image of the initial disodium glycyrrhizinate substance (magnification ×4).

The initial Na_2_GA powder consists of shapeless, polydisperse particles of an amorphous substance; the particles are transparent and beige in color (Figure A3).

**Figure A4:**
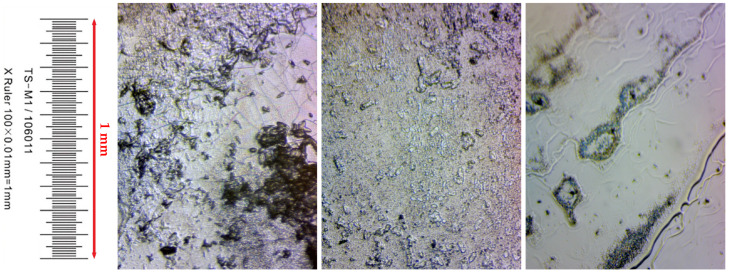
Microscopic image of recrystallized disodium glycyrrhizinate substance (magnification ×4).

Recrystallization of Na_2_GA from an alcoholic solution (under conditions analogous to those of SD synthesis by “solvent removal”) likewise does not affect the structure of the substance. The microcrystalline pattern is identical to that of the initial salt sample and is characterized by numerous shapeless, amorphous, polydisperse particles. In some micrographs obtained after recrystallization of Na_2_GA, a transparent, homogeneous Na_2_GA layer forms on the surface of the microscope slide, which cracks over time (Figure A4).

**Figure A5:**
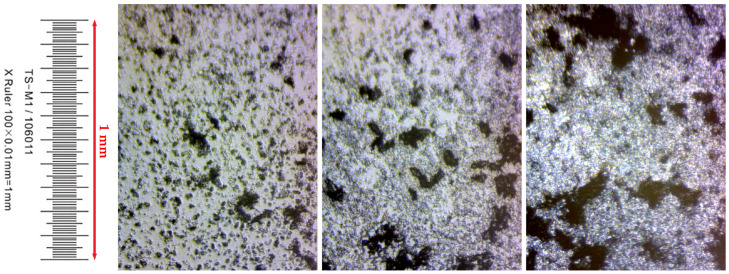
Microscopy of the recrystallized solid dispersion of leucomisine with Na_2_GA (1:5) (magnification ×4).

Solid dispersions of leucomisine with Na_2_GA (1:5) obtained by the “solvent removal” method resembles a superposition of the microcrystalline patterns of separately recrystallized leucomisine and Na_2_GA. It consists of a mixture of small, elongated, transparent crystals of recrystallized leucomisine and numerous fine, amorphous, shapeless particles of recrystallized Na_2_GA. Considering that the mass fraction of leucomisine in this SD is approximately 16.7% (based on a substance-to-carrier ratio of 1:5), the colorless transparent layer (background) is presumably a “solid solution” phase of leucomisine in Na_2_GA (Figure A5).

According to accumulated global experience, the study of various solid dispersion systems often includes different modifications of thermal methods. The purpose of such experiments is to determine the melting (thermal degradation) behavior of a substance incorporated into a carrier matrix, occurring during melting (or degradation) of the carrier. The main criterion for complex formation is the disappearance of thermal effects of substances incorporated into the matrix as an individual phase. In the present study, the investigation of leucomisine solid dispersions with Na_2_GA by differential scanning calorimetry (DSC) is of an auxiliary nature, as the experimental data obtained by microscopy are considered sufficient for the purposes of this work.

Visible-range spectroscopic data (Table 2) indirectly confirm the absence of any covalent interactions between leucomisine and Na_2_GA in the synthesized SDs. The spectral profile of leucomisine in SD solutions coincides with that observed for the solution of the parent leucomisine substance.

The microcrystalloscopic data allow the following assumption to be made: the leading factors responsible for the increased solubility of leucomisine from SDs obtained by the “solvent removal” method are micronization and partial loss of the crystalline structure of leucomisine during SD synthesis (i.e., prior to contact of the SD with water). At the SD synthesis stage (vacuum evaporation of ethanol), leucomisine partially dissolves in the Na_2_GA matrix, forming a solid solution.

In the literature [43,44,45], the formation of colloidal solutions is often indicated as one of the factors enhancing dissolution from SDs.

In this regard, the optical properties of SD solutions and their individual components were additionally investigated. During the dissolution kinetics experiments, all sample solutions appeared turbid. It was established that when filtered solutions (pore size 0.45 μm) of all investigated SDs (obtained by all methods described in this study) were illuminated with a narrow light beam, opalescence in the form of a bluish cone was observed—the Faraday–Tyndall effect—caused by the colloidally dispersed state of leucomisine present in these solutions (Appendix B). Similarly prepared solutions of Na_2_GA and the saturated solution of leucomisine substance did not exhibit the Faraday–Tyndall effect. The results obtained in this study thus confirm the presence of leucomisine in a colloidally dispersed state in SD solutions containing Na_2_GA.

To obtain reliable information regarding the physical state of leucomisine in its SDs prepared by the “solvent removal” method, X-Ray diffraction analysis of the studied samples was performed (Appendix C). The microcrystalloscopic data are fully consistent with the results of X-Ray phase analysis. The diffraction peaks observed in the leucomisine diffractogram indicate a pronounced crystalline structure of the substance. According to the Na_2_GA diffractogram, the crystalline structure of the SD carrier is less pronounced. The diffractogram of the leucomisine solid dispersion with Na_2_GA (1:5) obtained by the “solvent removal” method contains distinct peaks corresponding to leucomisine, indicating preservation of its original crystalline structure. Overall, the diffractogram of this SD is practically identical to that of the initial leucomisine substance.

IR spectrum (KBr, ν, cm^−1^) (leucomisine): 2937, 2919 (CH_3_), 2850 (CH_2_), 2864 (CH_3_), 1776 (C=O, γ-lactone), 1681, 1637, 1616 (cyclopentadienone), 1432 (CH_2_), 986 (C–H).

IR spectrum (KBr, ν, cm^−1^) (Na_2_GA): 3409, 3399, 3225 (OH), 2970, 2950, 2878 (CH_3_), 1709, 1652 (C=O), 1604 (C–O–C), 1402 (OH), 1047 (C–O–C).

IR spectrum (KBr, ν, cm^−1^) (leucomisine solid dispersion prepared by the solvent evaporation method, 1:5): 3418, 3399 (OH), 2977, 2941, 2876 (CH_3_), 1776 (C=O, γ-lactone), 1681, 1637, 1616 (cyclopentadienone), 1430 (OH), 1039, 986 (C–H).

In the IR spectrum of the parent leucomisine, absorption bands are observed at 1776 cm^−1^, characteristic of the γ-lactone carbonyl group, as well as at 1681, 1637, and 1616 cm^−1^, corresponding to cyclopentadienone vibrations. These bands are preserved in the IR spectrum of the leucomisine solid dispersion, indicating that the molecular structure of leucomisine remains intact in the solid dispersion. Furthermore, the absorption bands characteristic of Na_2_GA at 3418, 3399, and 1430 cm^−1^, associated with hydroxyl groups, are also present in the IR spectrum of the obtained solid dispersion.

The aqueous solubility of the resulting solid dispersion (SD) is primarily influenced by the SD preparation technology, as well as by the nature and ratio of its components. The enhancement of leucomisine solubility in SDs with Na_2_GA arises from a combination of factors.

During SD formation, the mechanism underlying the increase in aqueous solubility involves micronization, reduction in crystallinity, and partial amorphization of leucomisine, along with the formation of a solid solution of leucomisine within the carrier matrix, without alteration of its chemical structure. Upon dissolution of the SD in water, both true and colloidal solutions of leucomisine are formed.

The time-dependent fluctuations in leucomisine concentration observed in solutions of its SDs may represent the combined effect of opposing processes. On the one hand, the concentration increases due to the release of leucomisine from the SD matrix in a molecularly dispersed form (true solution) as well as in colloidal form. In this process, Na_2_GA acts as a solubilizer and a colloidal stabilizer, maintaining the previously achieved high concentration level. On the other hand, solution saturation enhances recrystallization of leucomisine molecules and coagulation of the colloidal particle fraction.

For different active pharmaceutical substances and SD carriers, the extent and contribution of these factors and processes to solubility are individual and difficult to predict. The balance of these processes upon reaching a steady leucomisine concentration in SD solutions is characterized by a complex dependence and represents the subject of a separate investigation.

It is likely that the superior effect on leucomisine solubility observed for SDs obtained by the “simple mixing” method (with leucomisine:Na_2_GA mass ratios of 1:5 and 1:7), compared with compositionally identical SDs obtained by the “solvent removal” method is directly related to their established microcrystalline structure.

Specifically, during dissolution of SDs prepared by “simple mixing”, recrystallization of the released leucomisine is initially minimal, and dissolution of its crystalline particles predominates. The colloidal solution of leucomisine forms after SD dissolution via a condensation pathway, as an incomplete recrystallization of a supersaturated solution. In this case, Na_2_GA solubilizes leucomisine molecules and protects its colloidal fraction.

In contrast, during dissolution of SDs obtained by “solvent removal”, due to the excessively rapid and abrupt (avalanche-like) release of leucomisine molecules from the Na_2_GA matrix (as well as the presence of a pre-formed colloidally dispersed leucomisine fraction, which most likely forms already at the SD preparation stage), recrystallization processes initially predominate. These processes prevent the leucomisine concentration from exceeding the levels achieved for SDs prepared by “simple mixing”.

This is clearly illustrated by the rapid clogging of syringe filter tips after each sampling step in experiments with “solvent removal” SD solutions, which exhibited a white turbidity with a slight grayish tint caused by the finely dispersed leucomisine phase. In contrast, such behavior was not observed for SDs prepared by “simple mixing”: their solutions were less turbid (the particle suspension was more coarsely dispersed), and a single filter was sufficient for the entire experiment comprising eight samples.

The developed solid dispersion based on leucomisine can be used as the active substance of an original hypolipidemic drug. The parent compound leucomisine exhibits hypolipidemic activity established in vivo in models of chronic alcoholism, alloxan-induced diabetes, acute hyperlipidemia, and fatty hepatosis. On its basis, the drug Aterolid was developed. According to the results of its clinical trials, it was found that in patients diagnosed with coronary heart disease or diabetes mellitus, therapy with Aterolid at a dose of 90 mg (three tablets) per day for two months leads to a reduction in cholesterol, triglycerides, and low-density lipoproteins. Administration of the new drug revealed no adverse effects [12].

The experimentally established increased water solubility of the solid dispersion of leucomisine and, consequently, its relatively high bioavailability will make it possible to reduce the leucomisine in the dosage form of a new hypolipidemic drug and to decrease the risk of side effects. Consequently, the hypolipidemic activity of solid dispersions based on leucomisine under in vivo conditions will be enhanced.

## 3. Materials and Methods

In this study, the sesquiterpene γ-lactone leucomisine (CAS No.: 68247-35-8) isolated from whitish wormwood (*Artemisia leucodes* Schrenk) was used; the substance met the requirements of the relevant regulatory documentation (JSC “Research and Production Center «Phytochemistry»”) [1,17].

Flower heads, buds, and leaves of *Artemisia leucodes* Schrenk were ground using a knife mill to a particle size ranging from 0.5 to 2 mm. A total of 6 kg of the milled leaves and flower heads were loaded into a UE-1 extractor (manufactured by Goro-Engineering LLC, Rostov-on-Don, Russia, under license from the Austrian company NATEX Prozesstechnologie GesmbH, Ternitz, Austria). Extraction of the plant material was carried out at a pressure of 160 bar, a temperature of 60 °C, for 3 h.

The yield of the CO_2_ extract from the plant material was 400 g (6.67% calculated on an air-dried raw material basis). The obtained extract, in the form of a viscous mass, was dissolved in ethanol, heated to 50 °C, and diluted with distilled water preheated to 70 °C at a solvent ratio of 2:1 (ethanol:water). The mixture was then kept in the dark and cooled to room temperature, resulting in the formation of a dark green coagulate. The precipitate (hydrophobic ballast substances such as lipids, chlorophylls, carotenoids, etc.) was separated from the ethanolic solution by vacuum filtration using a water-jet pump and a Büchner funnel with a paper filter. The collected precipitate was processed twice more following the same procedure.

The combined lactone fraction from the aqueous–ethanolic filtrate was extracted three times with chloroform at a ratio of 3:1 and subsequently evaporated. This procedure yielded a mixture of sesquiterpene lactones with a total mass of 267 g (4.45% calculated on an air-dried raw material basis).

The mixture of sesquiterpene lactones was transferred into a glass vessel and dissolved in a solvent mixture of heptane:acetonitrile:ethyl acetate (2:2:1) with a total volume of 1.8 L. The solution was thoroughly mixed and filtered through a folded filter. After filtration, the precipitate of the CO_2_ extract of *Artemisia leucodes* Schrenk was treated twice more with the same solvent mixture (heptane:acetonitrile:ethyl acetate, 2:2:1), using a mass-to-solvent ratio of 1:1.5. The combined filtrates were concentrated to a volume of 0.3 dm^3^ and then placed in a refrigerator for 1–2 h. The resulting precipitate was collected by filtration using a Büchner funnel. A technical powder was obtained, which, according to TLC analysis, contained a single individual compound.

The obtained powder was recrystallized from ethanol. A total of 41.4 g of the technical powder was placed into a round-bottom flask and dissolved in 0.7 dm^3^ of ethanol upon heating in a water bath at 65 °C. Ethanol was added gradually until complete dissolution of the powder was achieved. After dissolution, the flask was removed from the water bath and allowed to cool gradually (first at room temperature for 1 h, followed by cooling in a refrigerator at 2–5 °C for 1 h). During cooling, needle-shaped crystals progressively formed. The crystalline precipitate was filtered using a Büchner funnel and dried initially on filter paper under exhaust ventilation for 2 h. The material was then transferred to a porcelain dish and further dried in a Binder vacuum drying oven at 40 °C under a vacuum of 0.01 mbar until constant weight was achieved.

As a result, 36.6 g of a colorless crystalline substance (purity 99%) was isolated (0.61% calculated on an air-dried raw material basis and 13.71% relative to the total sesquiterpene lactones), corresponding to the molecular formula C_15_H_18_O_3_, with a melting point of 196–198 °C (EtOH) and an optical rotation of ${\left[\alpha \right]}_{D}^{20}$ + 56° (c 2.86, chloroform).

Leucomisine is a colorless crystalline compound with the molecular formula C_15_H_18_O_3_ and a melting point of 196–198 °C [17].

IR spectrum (KBr), ν (cm^−1^): 1771 (γ-lactone carbonyl), 1735, 1678, 1634, 1613 (cyclopentadienone).

UV spectrum (EtOH), λ_max_/nm: 256 (cyclopentadienone).

^1^H NMR and ^13^C NMR spectra are presented in Appendix D.

^1^H NMR (500.16 MHz, CDCl_3_, δ, ppm, J in Hz): 1.23 (3H, d, *J* = 7.0, H-13); 1.34 (1H, dd, *J* = 11.89, 11.67, H-8b); 1.94–1.90 (1H, m, H-7); 1.99–1.95 (1H, m, H-11); 2.24 (1H, dd, *J* = 6.87, 6.87, H-8a); 2.26 (3H, s, H-15); 2.28–2.26 (1H, m, H-9b); 2.31 (1H, d, *J* = 5.9, H-9a); 2.40 (3H, s, H-14); 3.38 (1H, d, *J* = 10.1, H-5); 3.59 (1H, t, *J* = 10.0, H-6); 6.13 (1H, br s, H-3).

^13^C NMR (125.76 MHz, CDCl_3_, δ, ppm): 12.39 (q, C-13); 19.95 (q, C-15); 21.74 (q, C-14); 26.03 (t, C-8); 37.61 (t, C-9); 41.21 (d, C-11); 52.61 (d, C-7); 56.40 (d, C-5); 84.28 (d, C-6); 131.91 (s, C-1); 135.63 (d, C-3); 152.31 (s, C-10); 170.11 (s, C-4); 177.75 (s, C-12); 196.09 (s, C-2).

Mass spectrum: HR-ESI-MS *m*/*z*: 247.1329 [M+H]^+^ (calcd for C_15_H_19_O_3_, 247.1329); MS/MS *m*/*z*: 201.1276, 191.1435, 173.0962, 159.0799, 158.0725 [46].

In the preparation of the solid dispersions, a derivative of a plant saponin, disodium glycyrrhizinate (Na_2_GA), was used as the carrier matrix. The compound was purchased from Shaanxi Sciphar Biotechnology Co., Ltd. (Xi’an, China; purity 98%). Na_2_GA (C_42_H_60_O_16_Na_2_) is a grayish-brown powder with a sublimation temperature of 400 °C.

**Preparation of Solid Dispersions.**

Based on an analysis of scientific and patent literature, three methods for the preparation of SDs with Na_2_GA were selected for further study, namely: “solvent removal”, “simple mixing”, and “mixture heating” [18,43,47].

**Solvent Removal Method.**

Portions of leucomisine (0.125 g) were dissolved in a minimal volume of 96% ethyl alcohol (analytical grade) at 80 ± 2 °C until complete dissolution. Subsequently, Na_2_GA was added in amounts of 0.250 g (1:2), 0.375 g (1:3), 0.625 g (1:5), or 0.875 g (1:7), followed by dissolution to homogeneity. The solvent was evaporated under vacuum to constant mass (from 0.375 g to 1.0 g). A UED-Lab115 vacuum pump (UED GROUP, Saint-Petersburg, Russia) and a UT-4301E bath (ULAB, Nizhniy Novgorod, Russia) were used [16,30].

**Simple Mixing Method.**

Calculated amounts of Na_2_GA (0.625 g for 1:5 and 0.875 g for 1:7) and leucomisine (0.125 g) were jointly triturated in a porcelain mortar for 1 min until a homogeneous mixture was obtained.

**Mixture Heating Method.**

Calculated amounts of Na_2_GA (0.625 g) and leucomisine (0.125 g) were mixed, and the resulting powder was heated at 120 °C for 30 min using a general-purpose laboratory drying oven LHT 5/60 (Carbolite-Gero, Neuhausen, Germany).

**Study of leucomisine Dissolution.**

The dissolution process was investigated according to the method described in [25]. The main challenge of the experiment was the inability to apply the method specified in the General Pharmacopoeial Monograph (GPM) 1.4.2.0014.15 to study the dissolution kinetics of the tested objects (powders and viscous masses), including issues related to obtaining saturated leucomisine solutions. The SDs studied were sticky, light-yellow, powder-like masses prone to agglomeration. Therefore, the conditions specified in GPM 1.4.2.0014.15 are not always suitable for studying the dissolution of these SDs. The rationale for the modified method is described in detail in our previous publications [22,25].

In this study, dissolution of the leucomisine samples was carried out using an RCT-BASIC thermostated magnetic stirrer (IKA, Staufen, Germany). Sample masses were selected to achieve maximally saturated leucomisine solutions during dissolution. Dissolution medium: 0.1 dm^3^ of purified water at 25 ± 1 °C. The studied samples were introduced into the dissolution medium and continuously stirred at 100 rpm. To evaluate the dissolution kinetics of leucomisine in water, 5 mL aliquots were withdrawn from the medium. Sampling was performed every 5 min (samples 1–4) and subsequently every 10 min until the end of the experiment (samples 5–8). After each withdrawal, the volume of the medium was replenished with 5 mL of purified water. The collected samples were filtered using syringe filter units (Minisart^®^, Sartorius, Göttingen, Germany) equipped with RC (regenerated cellulose) membranes with a pore size of 0.45 μm.

**Study of the dissolution kinetics of leucomisine.**

A UNICO-2800 spectrophotometer (UNICO (United Products and Instruments), Princeton, NJ, USA) and glass cuvettes with a 10 mm optical path length were used. The optical density of the filtrates was measured at the leucomisine absorption maximum of 256 ± 2 nm. Concentrations were determined using a calibration curve. The relative error of the mean values was approximately 4.57%.

**Microcrystalloscopy.**

A Levenhuk D50 LNG digital microscope (Levenhuk, Inc., Tampa, FL, USA) was used. The analysis was performed according to the methodology described in previous publications [25]. For the analysis of leucomisine substance, the powder was placed on a microscope slide, mixed with a drop of vaseline oil, covered with a coverslip, and examined microscopically. For SDs obtained by “solvent removal”, 3–5 drops of a solution of the SD components (Na_2_GA and leucomisine) in 96% ethyl alcohol, heated to 80 ± 2 °C and mixed in the appropriate ratios, were placed on a slide and examined after solvent evaporation. Recrystallized samples of leucomisine and Na_2_GA were examined separately using the same approach. After solvent evaporation and solidification, the dry residues were analyzed microscopically.

**X-Ray Phase Analysis.**

X-Ray diffraction was performed using a DRON-4 diffractometer (Burevestnik Production Association, Saint-Petersburg, Russia) with Co-Kα radiation. Data were collected over a 2θ range of 10–70°, with a step size of 0.05°, Bragg–Brentano geometry, and a counting time of 3 s per point.

The X-Ray phase analysis was carried out for a solid dispersion sample obtained by the solvent evaporation method with a substance-to-carrier ratio of 1:5.

Prior to the X-Ray analysis, the samples were placed in an agate mortar and ground with an agate pestle for 1 min.

Vaseline was then separately applied to the bottom and inner walls of the standard quartz cuvette.

The powdered sample was transferred from the agate mortar into the quartz cuvette (with vaseline) and compacted.

The cuvette was then placed into the instrument, and the measurement was performed.

**Investigation of Optical Properties of leucomisine.**

The optical characteristics of the obtained leucomisine solutions were studied according to the method described by Krasnyuk I.I. (Jr.) et al. [22]. A 30 mL chemical beaker (optical path length 25.0 mm) was filled with the filtered solution of the test sample. A sheet of opaque thick paper with a 1 mm diameter aperture was placed between the bottom of the beaker and the light source. A beam of white light was directed through the aperture into the solution, and the appearance of the beaker was recorded. Photographs were taken under both illuminated and darkened conditions using a Canon 5D Mark II camera (Canon Inc., Tokyo, Japan) (exposure time 20 s). The results are shown in Appendix B.

**Statistical Analysis.**

Statistical processing was performed in accordance with GPM 1.1.0013.15. The confidence interval limits for the mean leucomisine concentration in the studied solutions and the relative error were calculated. The boundary values of the confidence interval for the mean concentration of leucomisine in the studied solutions, as well as the relative error, were calculated.

## 4. Conclusions

The analysis of the experimental data presented above demonstrates that the preparation of solid dispersions (SDs) with a carrier such as Na_2_GA improves the dissolution of leucomisine in water in all cases. The stimulation of the leucomisine dissolution process from its SDs synthesized by the “simple mixing” method is more pronounced compared with the “solvent removal” and “mixture heating” methods. The enhancement of leucomisine solubility and dissolution rate (by approximately 19- and 100-fold, respectively) is associated with several factors, including the loss of crystalline state during SD synthesis, micronization, and the solubilization of leucomisine by the carrier during SD dissolution. In addition, the formation of a colloidal leucomisine solution stabilized by Na_2_GA contributes to the observed effects. The optimal SD composition (leucomisine:Na_2_GA) was determined to be not less than 1:5–7 by mass. The obtained results are intended to be applied in the development of effervescent dosage forms, specifically leucomisine granules and tablets, as formulations with enhanced bioavailability exhibiting antioxidant and hypolipidemic pharmacological effects.

## Figures and Tables

**Table 1:** Time-dependent changes in leucomisine concentration in solutions of the studied samples.

No.	Sample	Mass of Leucomisine: Na_2_GA (g)	Mean 2-oxo-5,7α,6,11β(H)-guaia-1(10),3(4)-dien-6,12-olide Concentration (×10^−2^ g/L); n = 5, *p* = 95%								Relative Error, %
			Sampling Time, min								
			5	10	15	20	30	40	50	60	
1	Leucomisine	0.125	0.904 ±0.072	1.604 ±0.114	2.453 ±0.147	3.767 ±0.193	4.349 ±0.175	4.602 ±0.139	4.602 ±0.93	4.602 ±0.50	4.55
2	SD (1:2) Solvent removal	0.125:0.250	32.770 ±2.622	32.434 ±2.270	32.349 ±1.967	32.264 ±1.613	32.178 ±1.326	32.093 ±0.963	32.008 ±0.659	31.923 ±0.364	4.55
3	SD (1:3) Solvent removal	0.125:0.375	49.250 ±4.004	53.550 ±3.781	50.280 ±3.017	47.610 ±2.400	47.530 ±1.953	47.460 ±1.457	47.440 ±0.991	47.440 ±0.517	4.57
4	SD (1:5) Solvent removal	0.125:0.625	57.773 ±4.685	64.868 ±4.612	66.383 ±4.003	66.909 ±3.372	67.597 ±2.731	68.079 ±2.042	68.234 ±1.440	69.181 ±0.768	4.57
5	SD (1:7) Solvent removal	0.125:0.875	67.175 ±5.475	71.119 ±4.985	69.793 ±4.195	67.730 ±3.387	67.437 ±2.785	67.145 ±2.075	66.852 ±1.384	66.559 ±0.667	4.56
6	SD (1:5) mixture heating	0.125:0.625	38.642 ±3.118	46.199 ±3.266	53.755 ±3.258	61.312 ±3.133	72.703 ±2.908	76.784 ±2.357	76.534 ±1.623	73.116 ±0.819	4.58
7	SD (1:5) simple mixing	0.125:0.625	77.499 ±6.246	91.628 ±6.469	87.099 ±5.304	88.804 ±4.511	88.189 ±3.607	87.495 ±2.695	86.358 ±1.857	85.446 ±0.966	4.59
8	SD (1:7) simple mixing	0.125:0.875	89.200 ±7.163	89.716 ±6.361	94.538 ±5.729	94.366 ±4.747	94.193 ±3.768	92.127 ±2.791	85.583 ±1.712	83.345 ±0.883	4.54

**Table 2:** Time-dependent changes in the optical density of solutions of the studied samples.

No.	Sample	Mass of Leucomisine: Na_2_GA (g)	Mean Optical Density of the Solution and Dilution Ratio with Water; n = 5, *p* = 95%								Relative Error, %
			Sampling Time, min								
			5	10	15	20	30	40	50	60	
1	Leucomisine	0.125	0.5248 (SF)	0.9319 (SF)	1.4244 (1:1)	2.1876 (1:3)	2.5256 (1:4)	2.6725 (1:5)	2.6725 (1:5)	2.6840 (1:5)	4.55
2	SD (1:2) Solvent removal	0.125:0.250	0.3806 (1:50)	0.3767 (1:50)	0.3584 (1:50)	0.3612 (1:50)	0.3725 (1:50)	0.3741 (1:50)	0.3646 (1:50)	0.3822 (1:50)	4.55
3	SD (1:3) Solvent removal	0.125:0.375	0.572 (1:50)	0.622 (1:50)	0.584 (1:50)	0.552 (1:50)	0.550 (1:50)	0.550 (1:50)	0.551 (1:50)	0.553 (1:50)	4.57
4	SD (1:5) Solvent removal	0.125:0.625	0.6710 (1:50)	0.7535 (1:50)	0.7710 (1:50)	0.7655 (1:50)	0.7907 (1:50)	0.7925 (1:50)	0.7851 (1:50)	0.8035 (1:50)	4.57
5	SD (1:7) Solvent removal	0.125:0.875	0.780 (1:50)	0.826 (1:50)	0.811 (1:50)	0.752 (1:50)	0.7676 (1:50)	0.7782 (1:50)	0.7376 (1:50)	0.777 (1:50)	4.56
6	SD (1:5) mixture heating	0.125:0.625	0.4488 (1:50)	0.4806 (1:50)	0.5070 (1:50)	0.7121 (1:50)	0.8444 (1:50)	0.8918 (1:50)	0.8889 (1:50)	0.8492 (1:50)	4.58
7	SD (1:5) simple mixing	0.125:0.625	0.900 (1:50)	1.064 (1:50)	1.011 (1:50)	1.031 (1:50)	0.9778 (1:50)	1.016 (1:50)	1.003 (1:50)	0.992 (1:50)	4.59
8	SD (1:7) simple mixing	0.125:0.875	1.036 (1:50)	1.042 (1:50)	1.098 (1:50)	1.096 (1:50)	1.094 (1:50)	1.070 (1:50)	0.994 (1:50)	0.968 (1:50)	4.54

**Figure A6:**
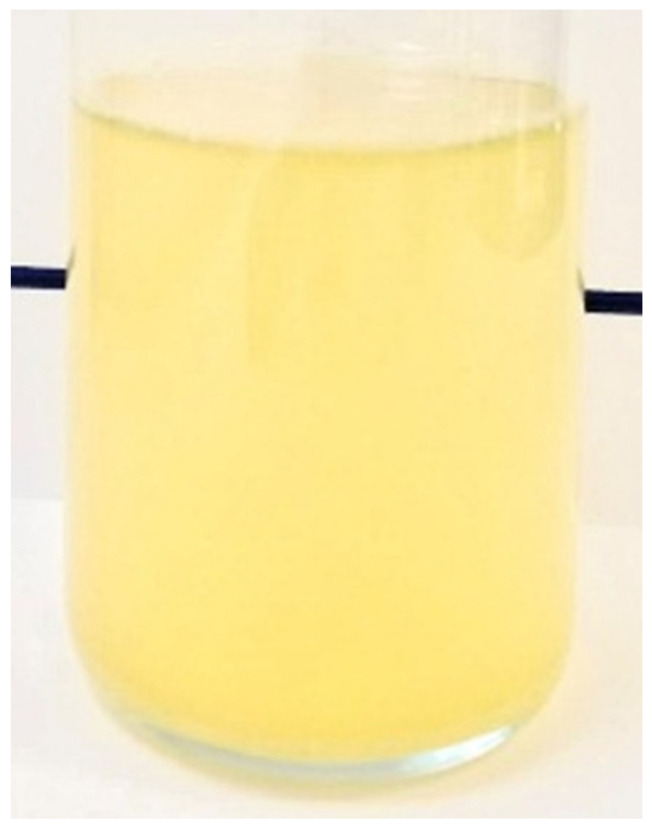
Results of the investigation of optical properties of solutions: appearance of a beaker containing the saturated solution of the leucomisine solid dispersion with Na_2_GA (1:2) obtained by the “solvent removal” method before filtration (leucomisine concentration: 31.923 × 10^−2^ g/dm^3^), turbid solution.

**Figure A7:**
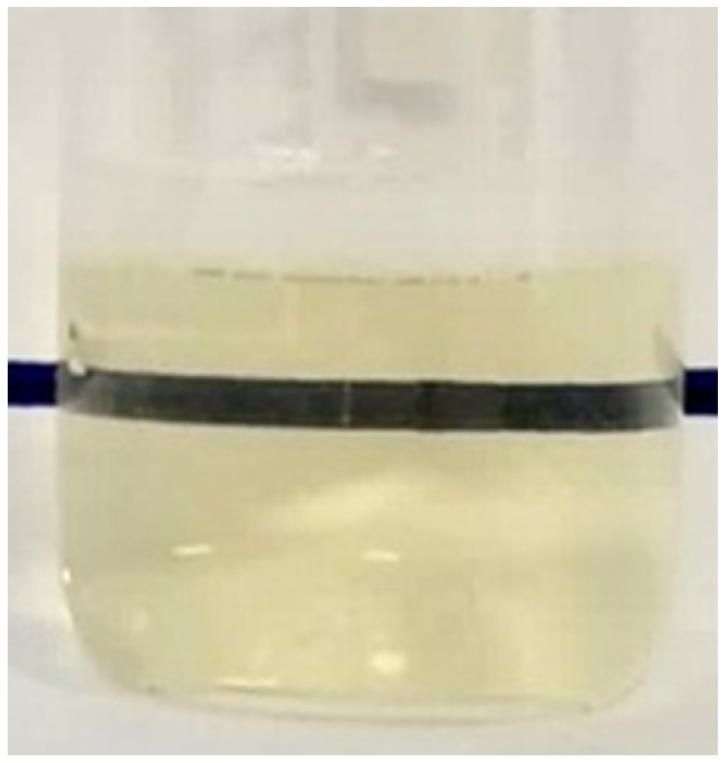
Results of the investigation of optical properties of solutions: the same solid dispersion solution after filtration (filter pore size 0.45 μm), transparent solution.

**Figure A8:**
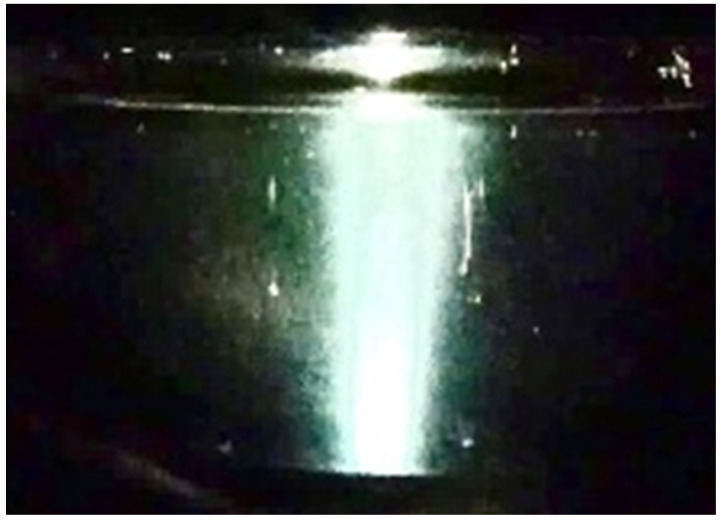
Results of the investigation of optical properties of solutions: Faraday–Tyndall effect (visible light cone indicated by an arrow) observed in the beaker containing the filtered solid dispersion solution; the photograph was taken under darkened conditions.

**Figure A9:**
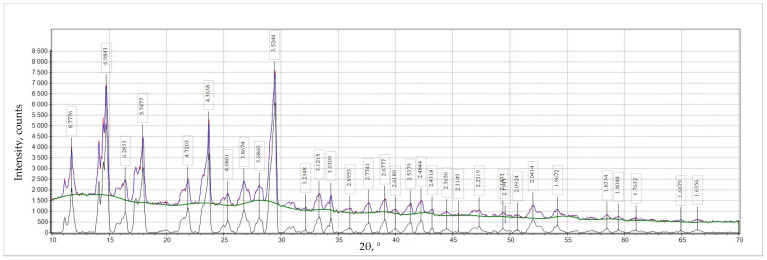
Powder X-Ray diffraction pattern of the parent leucomisine substance. Purple-red line—original X-Ray diffraction pattern of the sample; Green line—background line; Black line—processed graph (background subtracted from the original intensity).

**Figure A10:**
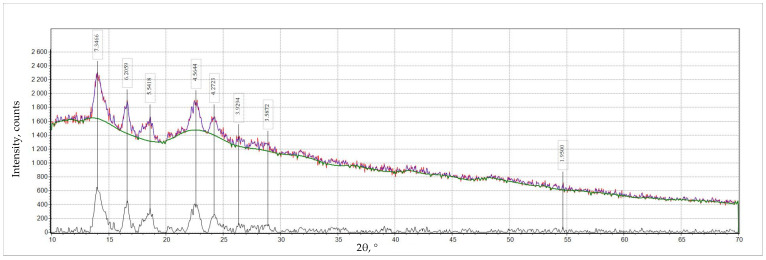
Powder X-Ray diffraction pattern of the Na_2_GA carrier substance. Purple-red line—original X-Ray diffraction pattern of the sample; Green line—background line; Black line—processed graph (background subtracted from the original intensity).

**Figure A11:**
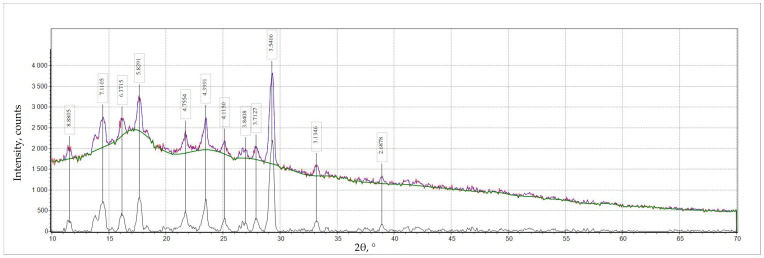
Powder X-Ray diffraction pattern of the leucomisine solid dispersion with Na_2_GA (1:5) prepared by the solvent evaporation method. Purple-red line—original X-Ray diffraction pattern of the sample; Green line—background line; Black line—processed graph (background subtracted from the original intensity).

**Figure A12:**
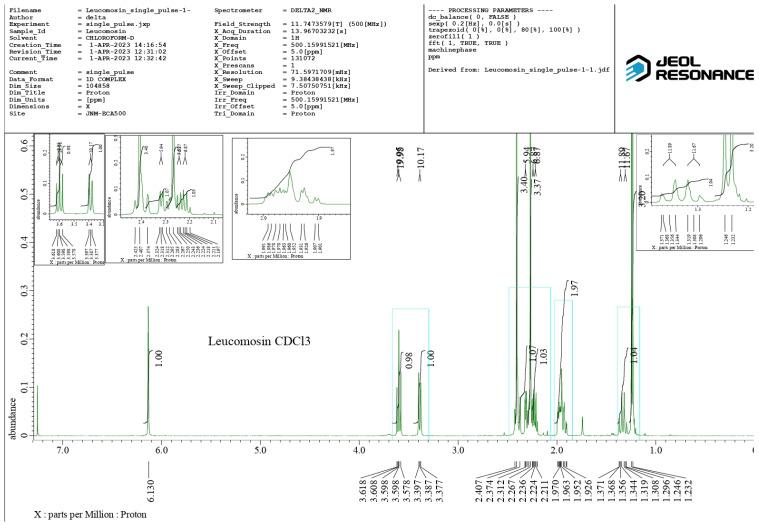
^1^H NMR spectrum of the parent leucomisine substance.

**Figure A13:**
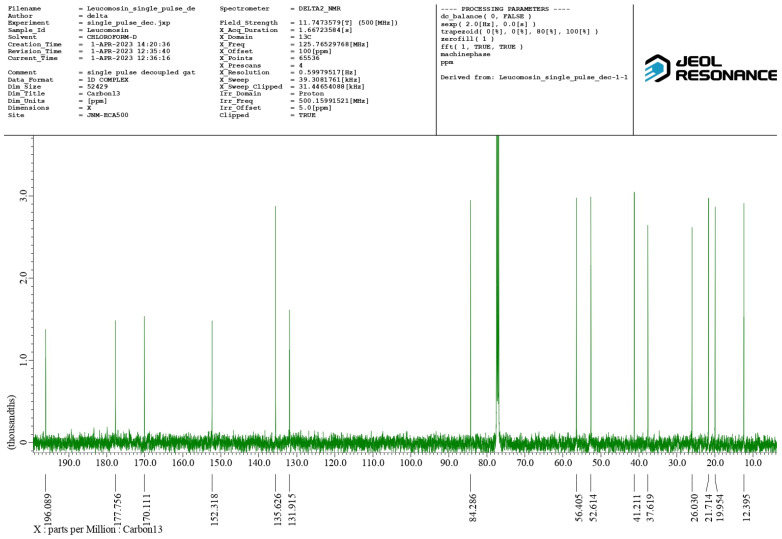
^13^C NMR spectrum of the parent leucomisine substance.

## Data Availability

The original contributions presented in this study are included in the article. Further inquiries can be directed to the corresponding authors.

## Abbreviations

The following abbreviations are used in this manuscript:

SD	Solid dispersion
Na_2_GA	Disodium glycyrrhizinate
GPM	General Pharmacopoeia article
DSC	Differential scanning calorimetry
SF	Solvent-free
